# US county-level agricultural crop production typology

**DOI:** 10.1186/s13104-019-4594-4

**Published:** 2019-08-30

**Authors:** Courtney R. Hammond Wagner, Meredith T. Niles, Eric D. Roy

**Affiliations:** 1 Rubenstein School for Environment and Natural Resources, University of Vermont, Burlington, Vermont USA; 2 Gund Institute for Environment, University of Vermont, Burlington, Vermont USA; 3 Department of Food and Nutrition Sciences & Food Systems Program, University of Vermont, Burlington, Vermont USA

**Keywords:** Agriculture, Farming systems, Typology, Crop production, United States, County-level

## Abstract

**Objectives:**

Crop production is an important variable in social, economic and environmental analyses. There is an abundance of crop data available for the United States, but we lack a typology of county-level crop production that accounts for production similarities in counties across the country. We fill this gap with a county-level classification of crop production with ten mutually exclusive categories across the contiguous United States.

**Data description:**

To create the typology we ran a cluster analysis on acreage data for 21 key crops from the United States Department of Agriculture’s 2012 Agricultural Census. Prior to clustering, we estimated undisclosed county acreage values, controlled for acreage in other crop types, and removed counties with low agricultural production to produce proportional scores for each crop type in each county. We used proportional scores to control for the influence of county size in the cluster analysis and used internal and stability measures to validate the analysis. The final dataset features 2922 counties. Future research can leverage this typology as an input for county- or regional-level analysis.

## Objective

Agricultural production is an important variable for many different types of analyses, including, for example, regional economic models, environmental assessments of water quality or greenhouse gas emissions, and analyses of social trends. While ample data are available to examine various aspects of agricultural production across the US, we lack a typology of recent agricultural production across the country. We present a dataset with an agricultural production typology for counties across the contiguous US, derived from a cluster analysis of the 2012 county-level crop data [1].

The US Department of Agriculture (USDA) Economic Research Service’s (ERS) Farm Resource Regions, defined in the early 2000s, are examples of agricultural regions partially based on county-level agricultural production [2]. The USDA uses the Farm Resource Regions to examine regional trends and determine program and funding priorities [3]. The ERS Farm Resource Regions were informed by a cluster analysis of county-level farming characteristics in the early 1990s [4]. Cropping patterns have shifted since 1991 due to economic trends, agricultural policy and climate change [5–8]. Using USDA crop data from the 2012 Agricultural Census, we provide a more up-to-date crop production typology.

The motivation for this dataset came from a need for a single categorical, county-level variable that incorporates the diversity of crop types grown throughout the US, where previous efforts were largely geographically focused. Additionally, we sought to capture similarities in agricultural production, including diversity of production and relative quantity of production, between counties. We anticipate that this dataset will be of use in analyses that seek to understand county-level patterns that may relate to crop production types, as we intend to do in a forthcoming publication focused on nitrogen dynamics.

## Data description

Our dataset identifies 10 mutually exclusive, agricultural crop production categories for the contiguous US based on crop production values in the 2012 USDA Agricultural Census Data [9]. Each crop production category is defined by the crops that emerged from the USDA crop data to drive membership in each county-level crop production cluster. These ten categories are: (1) corn silage and other crops, (2) tobacco, (3) hay, (4) barley, beans and sugar beets, (5) alfalfa and barley, (6) sorghum, sunflower and wheat, (7) oranges and sugarcane, (8) rice, (9) corn grain and soybeans, and (10) cotton and peanuts.

County-level crop acreage data was obtained from the USDA NASS Quick Stats Database from the US Agricultural Census of 2012 [9]. We downloaded county level crop acreage data for the 21 crops that, according to International Plant Nutrition Institute (IPNI), account for an average of 95% of harvested cropland acres reported in the agricultural census across the contiguous US [10]. The 21 crops are: apples, barley, canola, beans, corn grain, corn silage, cotton, alfalfa, oranges, peanuts, potatoes, rice, sorghum, soybeans, sugar beets, sugarcane, sunflower, sweet corn, tobacco, wheat and other hay (i.e. all hay acreage excluding alfalfa). Additionally, we downloaded the total county acreage and created a “22nd crop” which represents acreage of all other crops grown in the county that are unaccounted for in the 21 crops. The 22nd crop category captures acreage in the 55 crops included in the agricultural census that are less prevalent (i.e. combined represent only 5% of harvested cropland in the US), for example, cucumber, oats and cherries [9]. The initial data download from Quick Stats included 3060 counties, out of the total 3108 county equivalents in the contiguous US. The data cleaning process resulted in a final dataset of 2922 counties or 94% of the counties in the contiguous US.

USDA Agricultural Census data contains withheld data in the form of “(D)” and “(Z)” values in the dataset to avoid disclosing data for individual farms and to represent small figures, respectively [11]. To clean the dataset we changed all (Z) values to zero, as (Z) values represent a value of less than half an acre [11]. Then we followed the IPNI methodology [10] to estimate missing values for all (D)s in the dataset. We describe these methods in detail in the methods and technical validation document (see Table 1). We then created the 22nd crop variable by summing acreage for all 21 crops and subtracting this from the reported county total harvested cropland. To control for differences in county size, we transformed the absolute acreage values to proportional scores.

**Table 1:** Overview of data files/data sets

Label	Name of data file/data set	File types (file extension)	Data repository and identifier (DOI or accession number)
Data file 1	1_Crop_cluster_data_wrangling_forRepository_071919	R script (.R)	Figshare: 10.6084/m9.figshare.8132867.v2
Data file 2	2_Cluster_Analysis_22crops_forRepository	R script (.R)	Figshare: 10.6084/m9.figshare.8132867.v2
Data file 3	Methods_and_Technical_Validation_US_county_agricultural_clusters_figshare	Word file (.docx)	Figshare: 10.6084/m9.figshare.8132867.v2
Data set 1	crop_production_typology_data_and_metadata_052819	Excel file (.xlsx)	Figshare: 10.6084/m9.figshare.8132867.v2

We then performed a k-means cluster analysis [12] on the 22 county-level crop acreage proportional scores. We determined the optimal number of clusters, or crop production categories, to be 10. We analyzed the cluster scree plot, as well as ran internal and stability measures using the clValid R package [13]. We describe in detail the technical validation of our cluster analysis in the methods document (see Table 1).

## Limitations

We acknowledge that while we have justified and validated our selection of a 10-cluster solution, the selection of a different number of clusters would change the dataset.

## Data Availability

The datasets generated during the current study are publicly accessible in the Figshare repository [1]: 10.6084/m9.figshare.8132867.v2.

## Abbreviations

USDA: United State Department of Agriculture
ERS: Economic Research Service
IPNI: International Plant Nutrition Institute
NASS: National Agricultural Statistics Service
